# Reconstruction of nasopharyngeal defect with submental flap during surgery for nasopharyngeal malignant tumors

**DOI:** 10.3389/fsurg.2022.985752

**Published:** 2022-10-31

**Authors:** Hongzhi Ma, Jugao Fang, Qi Zhong, Lizhen Hou, Ling Feng, Shizhi He, Ru Wang, Yifan Yang

**Affiliations:** ^1^ Department of Otolaryngology-Head and Neck Surgery, Beijing Tongren Hospital, Capital Medical University, Beijing, China; ^2^ Key Laboratory of Otorhinolaryngology Head and Neck Surgery, Ministry of Education, Beijing Institute of Otorhinolaryngology, Beijing, China

**Keywords:** submental flap, nasopharyngeal carcinoma, defect repair, head and neck, surgery

## Abstract

**Objective:**

To investigate the feasibility and effect of a pedicled submental flap in postoperative defect repair of nasopharyngeal malignant tumors.

**Methods:**

Eight cases (six women, two men; age, 29–63 years) of postoperative defects after resection of malignant nasopharyngeal tumors with a lesion stage of (r) T_1–3_N_0–2_M_0_ were retrospectively analyzed. Preoperative enhanced thin-slice computed tomography of the neck was performed to predict the submental flap reflux vein. The submental flap was prefabricated during the operation, and the nasopharyngeal mass was removed through the parapharyngeal space approach combined with nasal endoscopy/mandibular external rotation/maxillary overturning. The submental flap was elevated to the nasopharyngeal defect area through the parapharyngeal space for repair.

**Results:**

Intraoperative examination confirmed that among the eight submental flaps, three had venous drainage into the internal jugular vein and five had venous drainage into the external jugular vein; these findings were consistent with the preoperative computed tomography findings. The size of the submental flap was 8–10 cm × 5–6 cm. The repair range reached the eustachian orifice on the healthy side and extended to the posterior wall of the maxillary sinus on the affected side. The flap extended to the posterior upper part of the nasal septum at the top, to the oropharynx at the bottom, and to the bony surface of the skull base at the deep side. Primary healing after surgery was achieved, and no flap necrosis occurred. After 3–77 months of follow-up, one patient with recurrent nasopharyngeal carcinoma after radiotherapy developed cervical lymph node recurrence again, one patient with adenoid cystic carcinoma had lung metastasis, and the remaining six patients had no recurrence.

**Conclusions:**

The pedicled submental flap is used to repair postoperative defects in the nasopharyngeal region through the cervical parapharyngeal space. It is a simple and fast procedure with adequate tissue volumes. The flap can effectively protect important structures such as the internal carotid artery and reduce the risk of infection and bleeding from postoperative wound exposure.

## 1. Introduction

The nasopharynx is hidden in the deep central craniofacial region. Malignant tumors in this region may invade the parapharyngeal space. In the past, nonsurgical treatment such as radiotherapy was recommended for nasopharyngeal carcinoma (1, 2). About 10%–40% of nasopharyngeal carcinomas recur after initial treatment with radiotherapy; among these, 20%–30% are rT1–rT2 lesions (3–6). The main treatment for recurrent nasopharyngeal carcinoma after radiotherapy is two courses of radiotherapy or palliative chemotherapy. If the local skull base bone and blood vessels are exposed after surgery in patients with recurrence of nasopharyngeal carcinoma after radiotherapy, intractable ulcers, local callus formation, necrosis, infection, and hemorrhage will often occur, seriously affecting patients’ quality of life. In severe cases, the infection may cause rupture of the internal carotid artery, leading to severe hemorrhage and death (7, 8). In recent years, with the advancement of surgical techniques, surgery has become the preferred option for patients with early- and mid-stage nasopharyngeal carcinoma that recurs after radiotherapy (9–13). Studies have shown that surgery is more effective than two courses of radiotherapy for recurrent early- and mid-stage nasopharyngeal carcinoma provided that complete resection can be performed (7). In addition, for some initially treated malignant nasopharyngeal tumors that are not sensitive to chemoradiotherapy, such as nasopharyngeal mucoepidermoid carcinoma and adenoid cystic carcinoma, radical surgical resection combined with postoperative radiotherapy is the most effective treatment modality. However, surgery often requires removal of the lateral wall of the nasopharynx and part of the skull base bone, resulting in a large postoperative defect that leads to exposure of important structures such as the carotid sheath and meninges to the nasopharynx.

Therefore, the defect requires an adequately sized tissue flap for repair and coverage. Because the nasopharynx is surrounded by bony structures, the only adjacent tissue that can be used for a flap is the nasal mucosa (14, 15). A nasal septal mucosal flap with the pedicle located in the anterior wall of the sphenoid sinus had the largest tissue volume; however, the blood supply of the nasal septal valve was easy to be affected by nasopharyngeal malignant invasion, preventing the flap from being used. If the nasal tissue flaps could be used, they are thin and relatively small, making these flaps unsuitable for the repair of large nasopharyngeal defects. Other repair materials such as temporalis muscle flap must extend from the temporal region to the nasopharynx, which is very traumatic (1, 16). Additionally, when using micro-vascularized free flaps, patients with nasopharyngeal carcinoma who have received head and neck chemoradiotherapy have a high risk of vascular anastomosis failure (17–19).

In recent years, we have carried out mandibular external rotation, maxillary overturning, or nasal endoscopy combined with a transcervical parapharyngeal space approach to remove nasopharyngeal tumors (20). We have found that repair and reconstruction with a local pedicled submental flap (hereafter simply referred to as submental flaps) on the nasopharynx through the parapharyngeal space is reliable. The advantages of less trauma, fewer complications, high success rate and good postoperative quality of life deserve further research and promotion, which have been summarized as follows.

## 2. Data and methods

### 2.1. Clinical data

The medical records of 8 patients with nasopharyngeal malignant tumors who underwent surgery in Beijing Tongren Hospital from November 2015 to April 2022 were retrospectively analyzed. The patients included 2 men and 6 women aged 29–63 years at the time of surgery. All tumors were pathologically confirmed before surgery (4 cases of recurrent nasopharyngeal carcinoma after radiotherapy, 2 cases of nasopharyngeal mucoepidermoid carcinoma, and 2 cases of nasopharyngeal adenoid cystic carcinoma). According to the 2017 American Joint Committee on Cancer criteria, the disease stage was (r) T_1–3_N_0–2_M_0_, including 2 cases of T3, 2 cases of T2, and 4 cases of rT2. Preoperative endoscopy and imaging showed that the lesions were confined to the parapharyngeal space and the medial side of the cervical sheath. There was no invasion of the internal carotid artery, masticatory muscle, cervical vertebrae, or intracranial or maxillary bones, and no distant metastasis was found. The patients’ general conditions were good, with an Eastern Cooperative Oncology Group score of 0 or 1. All eight patients underwent nasopharyngeal tumor resection through the transcervicopharyngeal space approach combined with nasal endoscopy/mandibular external rotation/maxillary overturning + local lymphatic dissection + submental flap repair under general anesthesia (Table 1). Three months after the operation, the Sino-Nasal Outcome Test-20 (SNOT-20) was conducted to evaluate the patients’ nasal function (21).

**Table 1:** Clinical data and treatments for 8 cases with malignant nasopharyngeal tumors.

Case	Age/sex	pTNM	Surg	Pathology	Flap reflux vein predicted by CT	Flap reflux vein in surgery	Flap size (cm)	Follow-up	Snot20 3 months after surgery
1	49/F	T2N0M0	A + B + C	Mucoepidermoid carcinoma	Internal jugular vein	Internal jugular vein	8 × 5	37 months/no recur.	7
2	48/F	rT2N0M0	A + B + C	Non-keratinizing squamous cell carcinoma	External jugular vein	External jugular vein	8 × 6	29 months/no recur.	6
3	55/M	rT2N0M0	A + B + C	Non-keratinizing squamous cell carcinoma	External jugular vein	External jugular vein	10 × 6	27 months/no recur.	6
4	37/F	rT2N2cM0	A + B + C + D	Non-keratinizing squamous cell carcinoma	External jugular vein	External jugular vein	8 × 5	25 months/recurrs in the 6th months	15
5	63/F	T2N0M0	A + B + C	Mucoepidermoid carcinoma	External jugular vein	External jugular vein	8 × 5	16 months/no recur.	5
6	53/F	rT2N0M0	A + B + C	Non-keratinizing squamous cell carcinoma	Internal jugular vein	Internal jugular vein	8 × 5	9 months/no recur.	7
7	29/F	T2N0M0	B + C + D + E	Adenoid cystic carcinoma	Internal jugular vein	Internal jugular vein	8 × 5	77 months/lung metastasis in the 4th year without recur.	18
8	63/M	T3N0M0	B + C + D + F	Adenoid cystic carcinoma	External jugular vein	External jugular vein	10 × 6	3 months/no recur.	20

A, nasopharyngeal tumor resection through the transcervicopharyngeal space approach combined with nasal endoscopy; B, local lymphatic dissection; C, submental flap repair; D, tracheostomy; E, nasopharyngeal tumor resection through the transcervicopharyngeal space approach combined with mandibular external rotation; F, nasopharyngeal tumor resection through the transcervicopharyngeal space approach combined with maxillary overturning; pTNM, pathological tumor-node-metastasis.

### 2.2. Surgical procedures

#### 2.2.1. Preoperative preparation

**Figure 1:**
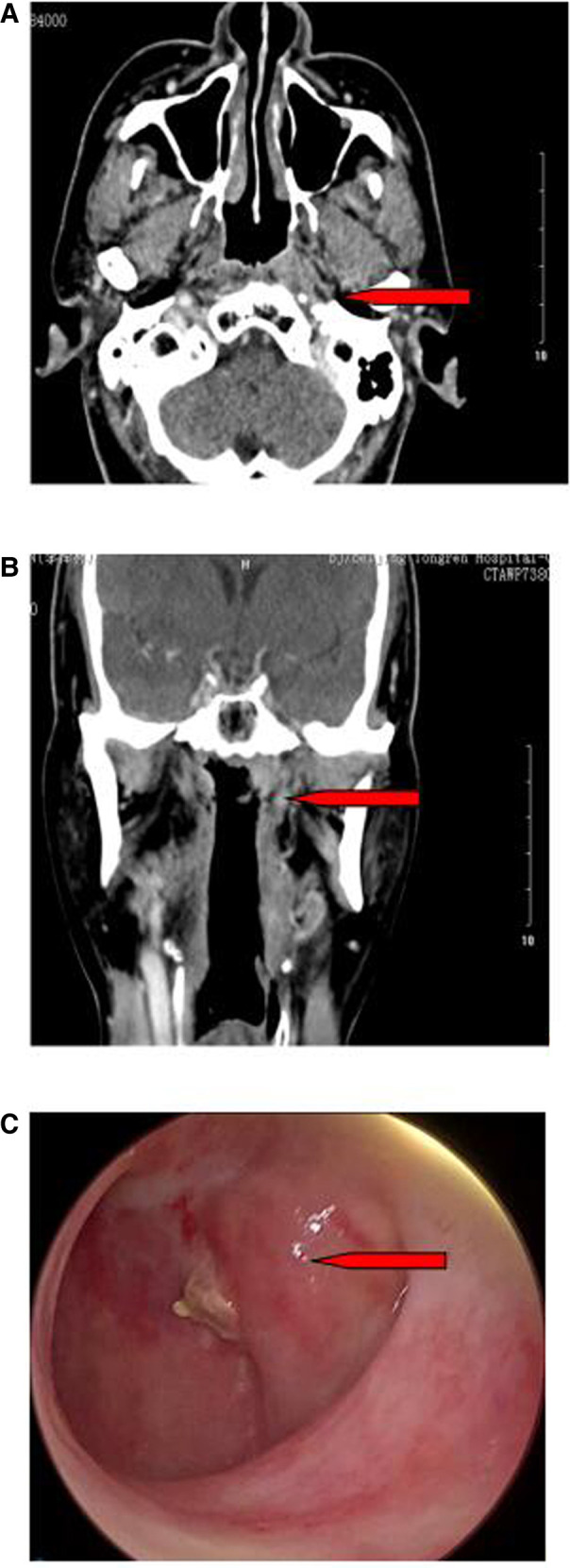
Picture and CT images for the nasopharyngeal tumor (arrows). (**A**) Axial CT Image. (**B**) Coronal CT. (**C**) Picture under nasal endoscopy.

Before surgery, all patients underwent enhanced nasopharyngeal computed tomography (CT)/magnetic resonance imaging (Figure 1), neck enhanced thin-slice CT (for predicting the reflux vessels of the submental flap), ultrasound of the cervical lymph nodes, teeth cleaning, nasal cleaning, evaluation of the whole-body condition, and screening for systemic metastasis. Male patients also underwent evaluation of the submental whisker distribution.

#### 2.2.2. Surgical steps

##### 2.2.2.1. Prefabrication of submental flaps

**Figure 2:**
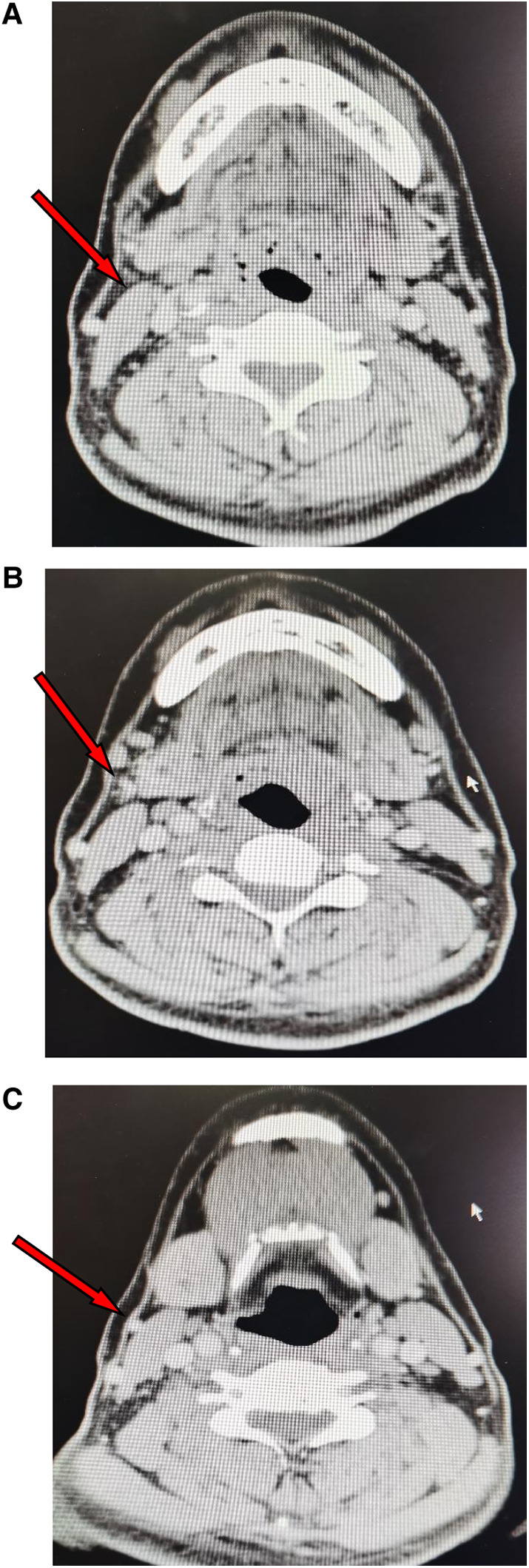
CT images showing the reflux vein of the submetal flap (arrows). (**A**) The reflux vein in the submandibular gland area. (**B**) The reflux vein drainaging into the external jugular vein. (**C**) The external jugular vein.

The upper margin of the flap was designed along the lower margin of the mandible (0.5 cm from the mandible lower margin), extending from the lateral side of the submandibular gland on the pedicle side to the mandibular angle on the opposite side of the pedicle. The lower margin of the flap was located at the thyroid cartilage (Adam's apple), and the flap measured 10–20 cm × 6–8 cm. According to the preoperative enhanced thin-slice CT of the neck, the submental flap reflux vein and its course (external jugular vein or internal jugular vein) were predicted (Figure 2).

**Figure 3:**
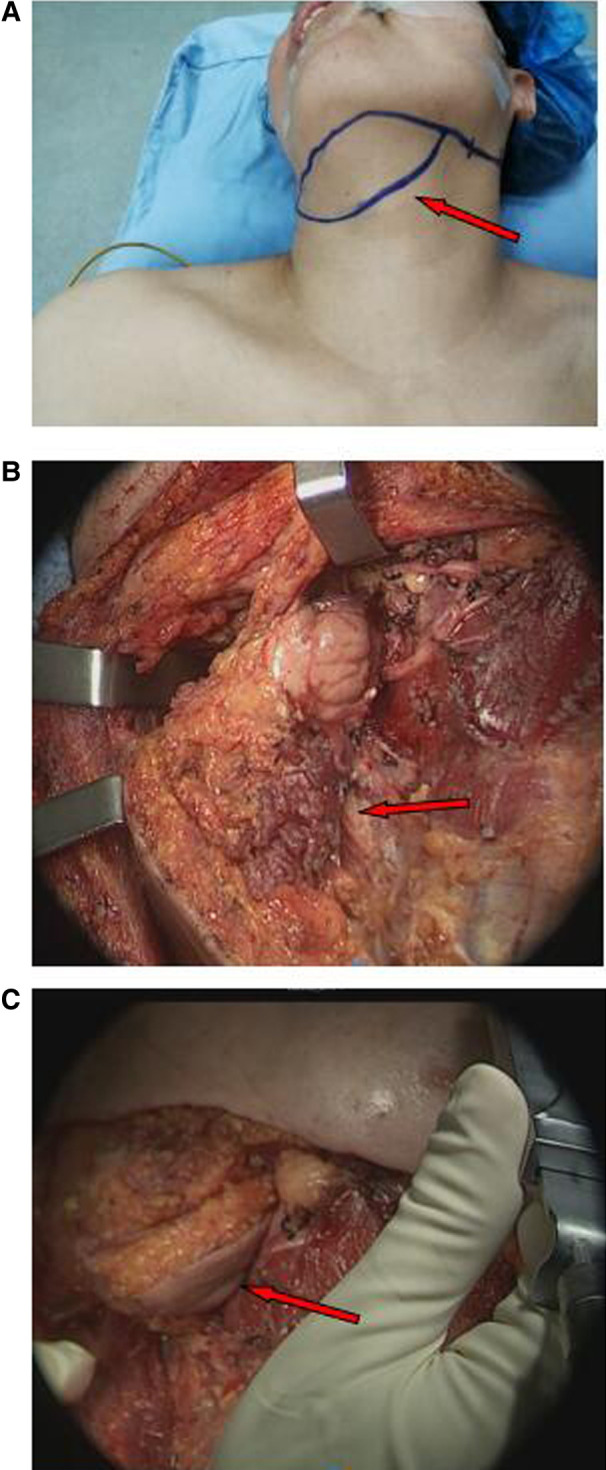
Reconstruction of nasopharyngeal defect with submental flaps (arrows). (**A**) Design of the submental flap. (**B**) Prefabrication of submental flap. (**C**) The submental flap was advanced into the nasopharynx through the cervical and parapharyngeal space.

According to the designed flap, the skin and platysma muscle was incised; and the marginal mandibular branch of the facial nerve was dissected and protected. The anterior belly of the digastric muscle of the affected side was detached at the chin to avoid separation of the anterior belly of the digastric muscle from the submental flap. The submental artery was found between the upper margin of the submandibular gland and the lower margin of the mandible, and the artery was protected at the submental flap side. According to the prediction based on preoperative CT, the refluxing vein was confirmed and then preserved in the submental flap. According to the relationship between the submandibular gland and the submental artery and reflux vein, the submandibular gland was either removed or retained at the base of the submental flap as appropriate. The flap was prefabricated for later use (Figure 3).

##### 2.2.2.2. Neck lymph node dissection

The neck lymph nodes were dissected according to the preoperative cervical lymph node ultrasound and cervical CT findings.

##### 2.2.2.3. Exposure of parapharyngeal space and lymph node dissection

The inferior portion of the parotid gland was elevated. The digastric muscle and styloid process muscle were then detached to identify the hypoglossal nerve and expose the parapharyngeal space. The carotid sheath was exposed, and dissection was performed along the surface of the carotid sheath to the cranial side. The blood vessels and important nerves at the base of the skull were dissected and protected, and the lymphoid tissues in the parapharyngeal space were dissected.

##### 2.2.2.4. Parapharyngeal space approach combined with nasal endoscopy/mandibular external rotation/maxillary overturning

Nasopharyngeal masses at the lateral, deep, and cranial sides were dissociated at the parapharyngeal space. Nasal endoscopy/mandibular external rotation/maxillary overturning was performed to thoroughly remove the masses (20). The scope of resection started from the lateral wall of the nasopharynx and the lateral wall of the sphenoid sinus for the healthy side; for the affected side, the scope included the torus tubarius, reaching the posterior wall of the maxillary sinus; and extending to the posterior and superior margin of the nasal septum at the top and to the oropharynx at the bottom. At the same time, the sphenoid sinus and sphenoidal rostrum bones were resected deep to the clivus bone at the skull base.

##### 2.2.2.5. Reconstruction of nasopharyngeal defect with submental flaps

After sufficient hemostasis, the epidermis at the proximal end of the preset submental flap was removed as appropriate to extend the pedicle, and the submental flap at the distal end was advanced into the nasopharynx through the cervical and parapharyngeal space. The flap was flattened to fully cover the defect of the nasopharynx and/or sphenoid sinus cavity. According to the positional relationship between the posterior nostril and the submental flap, two groups of suture traction lines at the lower part of the flap could be used to fix the submental flap to the nasopharynx. A moderately sized posterior nostril embolus was made with iodoform, and the submental flap was moderately compressed and fixed on the nasopharynx.

##### 2.2.2.6. Suture area

A negative-pressure drainage tube was placed in the parapharyngeal space and neck, and the subcutaneous skin was sutured layer by layer.

### 2.3. Postoperative treatment

Neck bracing with slight forward-leaning was performed. Compression on the submental flap pedicle was forbidden. Attention was given to oral hygiene, and anti-inflammatory, symptomatic treatment was administered.

The postoperative SNOT-20 score (including runny nose, sneezing, cough, ear discomfort, headache and psychological problems) was investgated 3 months after the surgery to evaluate the influnces caused by surgery.

## 3. Results

**Figure 4:**
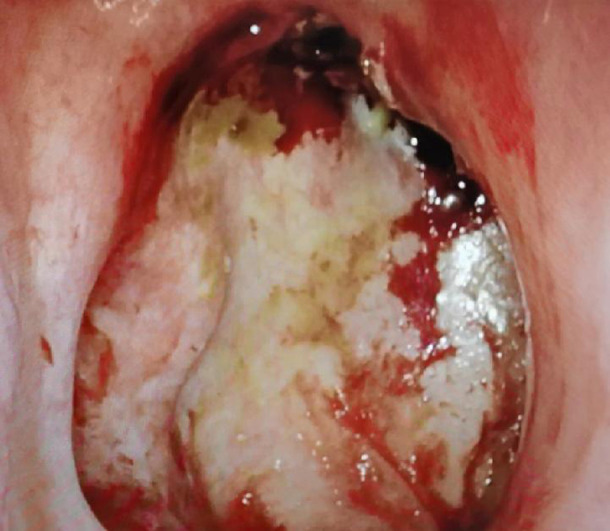
Picture of the submental flap 27 months after the operation.

All eight patients had negative incisal margins. The average blood loss was 280 ml, and the average operation time was 7.5 h. The patients were discharged from the hospital 5–7 days after the operation. The nasal packing was removed 10–14 days after the operation. The average size of the submental flap used to repair the nasopharyngeal defects was 8–10 cm × 5–6 cm, and all flaps survived after surgery. Intraoperative examination confirmed that all eight patients had venous drainage of the submental flap (three with venous drainage into the internal jugular vein and five with venous drainage into the external jugular vein); these findings were consistent with the prediction by preoperative cervical enhanced CT. ALL patients had nasal obstruction and poor nasal ventilation due to postoperative swelling of the submental flaps. 3 to 6 months after the operation, the nasopharyngeal submental flap gradually shrank and became mucous membrane with good ventilation. In the nasal endoscopy group, no obvious ulcer surface or dry scab formation was present (Figure 4). The SNOT-20 was administered 3 months postoperatively, and the score in the nasal endoscopy group was significantly lower than that in the mandibular external rotation group and maxillary overturning group. The patients’ quality of life after nasal endoscopy was good (Table 1).

During the follow-up period of 3–77 months, four patients underwent supplemental radiotherapy, two underwent chemotherapy. Three patients underwent tracheotomy and the cannula was smoothly removed 2 weeks after the operation. In one patient who developed recurrence of nasopharyngeal carcinoma after radiotherapy (rT2N2cM0), bilateral cervical lymph node metastasis occurred simutaniously; cervical lymph node recurrence occurred again 6 months after the last operation. Another patient had lung metastasis without nasopharyngeal recurrence 4 years after surgical treatment of nasopharyngeal adenoid cystic carcinoma. Both patients underwent immunotherapy. The remaining six patients had no recurrence.

## 4. Discussion

The wound after excision of malignant nasopharyngeal masses is large, and opinions differ on how to treat postoperative wound defects (including no repair, nasal mucosa flap repair, and temporalis muscle and free flap repair). Submental flaps were used to repair postoperative defects in patients with malignant nasopharyngeal tumors in this study. To the best of our knowledge, the use of these flaps in malignant nasopharyngeal tumors has never been reported. In this study, after resection of malignant nasopharyngeal tumors, the nasopharyngeal defect was 6–8 cm × 4–5 cm in size; For radical resection of nasopharyngeal masses, we believe that most of the parapharyngeal constrictor muscles need to be removed to avoid residual mass caused by difficult identification of the tumor and post-radiotherapy scar tissues. This will ensure the greatest extent of tumor resection and achieve negative incisal margins, minimizing the recurrence rate.

The submental flap was taken from the submental area, and the skin in this area was loose in middle-aged and elderly patients. The length of the flap could reach the bilateral mandibular angle (20 cm), the width could extend from the lower margin of the mandible to the superior margin of the thyroid cartilage (7–8 cm), and the depth could reach the platysma and anterior belly of the digastric muscle. In addition, a portion of the suprahyoid muscles and submandibular glands could be retained in the submental flap as appropriate, facilitating repair of large-scale defects in the middle skull base; the tissue volume in the submental flap could be adjusted and the thickness was moderate, preventing blockage of the nasal cavity and detrimental effects on postoperative ventilation. The submental flap was closer to the nasopharynx than the pectoralis major, and the pedicle reaching the nasopharynx was long enough to maintain a relatively stable blood supply. In this study, the size of the submental flap was at least 1 cm larger than the actual defect diameter, allowing the flap to fully cover the wound surface. The submental flap was adjacent to the nasopharynx but located at a safe distance, which was hardly invaded by the malignant tumors. Compared with nasal mucosa flaps, which have a small tissue volume and easily damaged blood supply, submental flaps are an ideal and reliable repair material for nasopharyngeal defects.

We have repaired postoperative defects of nasopharyngeal carcinoma using temporalis muscle, but the surgery was highly traumatic and associated with many postoperative complications. Different incisions are used for the temporalis muscle flap area and the neck dissection. After the temporalis muscle flap is taken, the flap area is sunken, affecting the appearance; this is more obvious for patients with thinning hair, especially after radiotherapy and chemotherapy. Many patients with cancer are old and weak, and their temporalis muscle is thin; it cannot adequately cover the nasopharynx, leading to bone exposure of the nasopharynx after surgery, long-term ulceration, scabbing, and infection (1, 22). We adopted the approach through the parapharyngeal space to push the submental flap up to the nasopharynx; this distance is short, and the operation is easy. The same incision is used for the submental flap donor site, neck lymph node dissection, and parapharyngeal space lymph node dissection, making the procedure more convenient and quicker than temporalis muscle flap prefabrication and with less collateral injury. The submental flap with the overlying skin can be used to repair the nasopharynx, unlike the temporalis muscle, which requires secondary epithelization. Free vascularized flaps (e.g., forearm flaps, medial calf flaps) can also be used to repair nasopharyngeal defects, but they require vascular anastomosis and have a long operative time (23). In addition, most patients have a history of radiotherapy in the neck, resulting in slender and poorly elastic blood vessels that make anastomosis difficult and result in treatment failure. The survival rate of free flaps is lower than that of submental flaps, and free flaps are more complicated to design. After repair with the submental flap, the temporalis muscle and the vessels of the neck recipient area for free flaps are retained, providing a backup plan for postoperative repair when the tumor recurs again. Of course undesirable hair growth at the recipient site can be asignificant concern (23, 24). When designing a submental flap, the course of the submental artery for the submental flap blood supply is relatively constant, but the course of the reflux vein is uncertain. Failure of the submental flap is mainly attributed to injury to the reflux vein. According to the relevant literature, the reflux vein is usually either the external jugular vein or the internal jugular vein (25). We propose prediction of the submental flap reflux vein and its course using preoperative neck enhanced continuous thin-slice CT followed by intraoperative confirmation that the submental flap reflux vein is completely consistent with the CT-based prediction (7). Through CT prediction, the submental flap reflux vein can be fully protected in advance, which greatly improves the survival rate of the submental flap. In this study, repair with a submental flap was successful in all eight patients; no local necrosis, infection, or bleeding occurred; For the experienced surgeon, failure rate of submental flap repair has been very low and mostly related to rare technical intraoperative errors (24). The submental flap was of moderate thickness, and the posterior nostrils became blocked to varying degrees in a short period of time after the operation, resulting in poor nasal breathing. After about 3–6 months, the submental flap gradually shrank, and the nasal cavity normalized, restoring normal ventilation. The postoperative SNOT-20 score of six patients who underwent endoscopy was significantly lower than that of two patients who underwent mandibular external rotation or maxillary overturning. It is suggested that the nasal function of the former is significantly better than that of the latter, although it cannot reach statistical significance due to the small number of cases.

## 5. Limitations

As a retrospective study, this study only involved 8 patients, with a small sample size and a short follow-up time of only 3–77 months. All nasopharyngeal lesions were (r)T_1–3_ (early- and mid-stage lesions). However, the clinical experience accumulated from such cases can be used to gradually expand the surgical indications. The two men in this study had inconspicuous submental whiskers, making nasopharyngeal repair suitable. For men with thick submental whiskers who undergo nasopharyngeal repair with a submental flap, nasopharyngeal hair removal is required in the later stage.

## 6. Conclusion

In conclusion, postoperative defect repair of nasopharyngeal malignant tumors with a submental flap through the parapharyngeal space is a simple and feasible approach with sufficient tissue volume, minimal trauma, and a high success rate. It thus resolves the difficulty of repairing large wounds in the nasopharynx and reduces postoperative complications. This method is reliable and effective for postoperative defect repair of most malignant nasopharyngeal tumors, and it is worthy of widespread promotion. Preoperative CT is used to examine the submental flap reflux vein and predict its course, facilitating easier harvesting of the submental flap and ensuring its survival.

## Data Availability

The original contributions presented in the study are included in the article/Supplementary Material, further inquiries can be directed to the corresponding author/s.
